# The Late Meeting

**Published:** 1889-05

**Authors:** 


F. E. DANIEL, M. D., EDITOR AND PUBLISHER.

THIS JOURNAL, by unanimous vote of the members, was made the Official Organ of the Austin District Medical Society.

COLLABORATORS:

*Dr. R. M. Swearingen, Austin.*
*Prof. B. E. Hadra, M. D., Galveston.*
*Prof. Geo. Cupples, M. D., San Antonio.*
*Dr. T. C. Osborn, Cleburne, Texas.*
*Dr E. J. Doering, Chicago.*
*Dr. E. J. Beall, Fort Worth.*
*Dr Odo Betz, Germany.*
*Dr. J. W. McLaughlin, Austin.*
*Dr. T. J. Tyner, Austin.*
*Prof. J. F. Y. Paine, M. D., Galveston.*
*Dr. R. H. L. Bibb, Mexico.*
*Dr. T. J. Bennett, Austin.*
*Dr. Bat Smith, Wharton.*
*Dr. E. Meierhof, New York.*
*Prof. W. B. Rogers, M. D., Memphis, Tenn.*

### THE LATE MEETING.

We believe we express the general sentiment when we say that the late meeting of the Texas State Medical Association, at San Antonio, was one of the best, and most satisfactory, within the recent history of the organization. It was one which will long be remembered for the quantity and quality of scientific work done, and real progress made. It was entirely, harmonious, and the best of feeling prevailed. Content, and a desire to please and be pleased, seemed to characterize the members present. All came, determined to have a good time, and a good time they had; not only scientifically, but socially. For once at least, business was not subordinated to pleasure, although it was observed that many chairs were empty during routine work, or the reading of some unusually long and prosy paper, and,—who could blame those members, mostly young men,—or, those who had never before seen the enchanted city? whose every street and wall could "a tale unfold, of deepest darkest woe"—perhaps—at any rate—a tale; for the "fields were so green, and the skies were so blue," the rose gardens so redolent of the perfume of spring,—the sparkling little river, winding like a silver thread through the quaint old city,—crossing and being crossed by every street and alley; sparkling in the sunlight of those glorious April mornings, as its waters splashed to the dip of a vagrant oar, or sparkled darkly beneath the moss grown and willow clad banks. Who could blame them, if, for a brief hour, they stole away from recital of "interesting cases" and preferred the notes of the song bird to "clinical notes;" the perfume of the flowers to the oft-told tale of "lacerated perinei," or Emmet's operations? The sights and sounds in a strange and beautiful Southern metropolis—the dark-eyed and willowy tropical beauties —gay in Easter finery,—or the pretty shop-windows,—or a drive to San Pedro Springs, and a look at the tame fishes sporting in the crystal waters, were indeed enticing; more so than the most elaborate "report" that could be read. Ah me!—"all work and no play, makes Jack a dull boy."

But we digress. We may say, science and social enjoyment were so equally blended—and each in its time and place—that it would be hard to say which predominated, if either. And then, the delightful hospitality of those real Southern people! But we mnst condense—for space is space.

All returned to their homes—the better for the meeting; carrying pleasing recollections of the occasion, and determined, never, no never, again to be absent from a single annual "gathering of the clan," all intending to tell their stay-at-home friends how *they* missed it; all convinced and gladly—that the old mother association had partaken anew of the elixir of life, and taken on a new lease. She is again—one and indivisible! No more now of sectional strife or jealousy; no division of sentiment. The Goddess of Harmony has driven out Discord; the cracks are closed, the crevices cemented—and we are again, as of yore—a band of brothers!

The courtly Paine made a splendid presiding officer, and if "all" did not go "as merry as a marriage bell," it went smoothly, at least. Business was dispatched in a most satisfactory manner, and everything was done in its time and place.

Of the results, we cannot now speak at length. The papers were mostly good, some excellent, and the plan of conducting the sections singly, as advised by the JOURNAL, proved much more satisfactory than the simultaneous sittings instituted at Galveston. It enabled all the members (who wished to) to hear all papers and all discussions. The papers and the discussion—the greater part of which was secured through the courtesy of the Express stenographic reporter, will make a splendid volume of Transactions.

The election of Dr. Swearingen to the Presidency was eminently satisfactory. His popularity is something truly extraordinary, and is only equaled by his real worth and ability. It gave rise to unbounded enthusiasm, and all feel and believe that he can do much to advance the interests of organized medicine. He is no sectional man, but the representative at large of the profession of the State, endorsed by all. He is a *born* parliamentarian, as well as a born orator. The *idol* of his friends, he is admired and respected by all. He is a man of wonderful personal magnetism, and the profession may well feel secure in the assurance that the interests of medical science and of the State Association will not suffer in his keeping.

The choice of Vice Presidents was no less happy. Sims of Northwest Texas, Kingsley of Southwest Texas, and Clark of the "free State of Schulenburg," all popular favorites, constitute a pleasing setting to the chief gem in the galaxy of officers. By-the-bye, the same handsome engraving of our President that adorns these pages will be inserted in the Transactions, that Dr. Swearingen's many friends may secure a copy, as a souvenir of this happy meeting.

Of the work done, perhaps the most important step was the action of the Convention in instructing the President and Secretary to apply at once to the Secretary of State for a charter! This the JOURNAL has advocated and worked for for years, and its accomplishment is in large part due to the Austin District Medical Society, which, at its last meeting, by resolution, instructed its delegates to this Convention to use every effort to secure said action; and to our *confrere*, Dr. T. J. Bennett, especially, is due the credit for pushing the subject, which, in the multiplicity of others, would, perhaps, have been overlooked, but for his zeal.

The President and Secretary will take great pains in drawing up the charter; its importance could hardly be overestimated when we reflect that it is the very first step in the direction of recognition; that the taunt has been thrown in the face of the profession that they have "no official status." Well—now, they have official status, and certain carping papers may lay to their souls the very consoling assurance that the profession will make the most of it.

Of the very important resolutions introduced by delegate Swearingen from the Austin District Medical Society, and which are published in full in the synopsis of proceedings herewith given, it is hardly necessary to speak—they speak for themselves. So fully do they echo the general sentiment, they were unanimously adopted by rising vote. Suffice to say, these resolutions will be printed and a copy placed in the desk of every delegate to the big Convention of Railway Surgeons, shortly to meet in St. Louis, and we look for much good to come out of it.

The impression prevails that the Committee on Medical Legislation have been idle; have done nothing. A careful perusal of the elaborate and very able report of the committee published in the proceedings herewith, will disabuse any unprejudiced persons of such notion, and will convince them that any direct appeal to the Legislature for the passage of any bill, in the existing state of public sentiment, would have been as unwise as it would have been useless. Chairman Cupples points out the obstacles in the way, and to be overcome before we can reasonably hope for such legislation as is desired and needed. The committee, it will be seen, have a well digested and deliberate plan of campaign mapped out, and they will work it out to a successful issue if given time and the necessary aid from the profession. But, in our judgment, it will be necessary as a part of the plan, to have an intelligent sub-committee of able and representative men on hand at the Capital, to press the matter; and it will take money to do this. This brings up another important topic. The members of the State Association are, many of them, shamefully delinquent in the matter of payment of their dues; and it is a matter of vital importance that they should pay up. Bills will shortly be sent to all who are delinquent, and the Secretary will be authorized by the President to draw at sight on such as are accessible through the banks. It is a matter of fact that many in attendance at San Antonio failed to register, and consequently, failed to pay their dues. Of the estimated three hundred and fifty present, (at least that number were decorated with the association badge, and had all the privileges of membership,) only one hundred and seventy-six registered and paid dues; and of this number, sixty were new members, who paid $10 each, (initiation fee and a year's dues.) This leaves then, one hundred and sixteen of the old members out of estimated two hundred and ninety who paid any money at all. These are facts, and it is not at all creditable to said members.

The association now carries on its roll, the names of five hundred and two active members. Each owes the association, and should pay $5. This, without estimating the receipts from new members, which at San Antonio alone was six hundred dollars, and the per capita tax of 50c. on each local society represented, should put into the treasury each year, $2500; an amount amply sufficient for all legitimate purposes.

In order to secure the payment of all dues at the next meeting, the committee of arrangements should, and doubtless will be instructed by the President to *place the badges in the hands of the Treasurer*, as is done by the American Medical Association, to be given to members only on the payment of a years dues, and handed over along with a receipt. This would also secure a correct and complete registration of members present. All will remember what confusion arose from want of a complete record at the late meeting.

The repeal of the prize essay ordinance was a judicious step, and met with hearty approval.

As advised by the JOURNAL, the proposed new Constitution and By-laws was not adopted ; and in its stead a few much needed and very sensible amendments were made, as will be seen by reference to the report of the proceedings published in this issue.

Of the social features of the occasion, we will not speak. We have not room to do justice to the subject; but suffice it to say, the entertainment was delightful, gratefully enjoyed, and highly creditable to the local committee;—twenty-seven hundred dollars was the sum expended by the committee. The banquet was grand; and if a flaw could be picked in that connection, it would be to say that the embellishment of the menu card with a skeleton was entirely inappropriate; there was no "skeleton" at that feast, at least. It has been suggested that it was intended *figuratively* to stand for, and represent the alleged "raw oysters on half shell" and "boned turkey" which the *menu* (by the bye, it was distributed *after* supper), called for,—but some declare that not the ghost of an "oyster," nor the skeleton of a "*boned*" *turkey* did *they* see, and they had on their specs long before the wine begun to circulate. We wish we *could* give an account of that banquet; of the *toasts* and responses, at least; they were "rich, rare and racy;" and the speech of our President-elect, was, especially, graceful and pleasing.

Long will the scenes and doings of this gathering linger in the chamber of memory of all who had the good fortune to be present.

